# I Can't Get No (Boolean) Satisfaction: A Reply to Barrett et al. (2015)

**DOI:** 10.3389/fpsyg.2016.01880

**Published:** 2016-12-06

**Authors:** Robert King

**Affiliations:** School of Applied Psychology, University College CorkCork, Ireland

**Keywords:** evolutionary psychology, extended mind, cognition, embodied cognition, artificial intelligence

Sometimes history can be philosophically interesting. Barrett (2011) and colleagues (e.g., Barrett et al., 2014, 2015) are to be congratulated on widening the scope of our understanding of animal cognition to include its ecological elements. However, in their eagerness to overturn a narrow model of computation, she and her colleagues have glossed over some rather interesting and salient historical facts. This is poignant, as these facts strengthen their case, and sharpen the focus on the more complete picture of ethologically valid cognition that they are drawing.

The key figure missing from the usual historical narrative is George Boole, whose bi-centenary has just passed and (it just so happens) is the luminary whose soon-to-be-restored home is visible from the office where I type this, in the University he led, and on the machine that his insights made possible.

Barrett (2011) wants to draw a distinction between computation—in a narrow sense–abstracted from any particular setting, and the highly embodied—especially ecologically rooted–cognition that she sees in the animals she studies.

In support of this distinction, she cites Searle's (1990) claim that, as a matter of history, humans tend to use their most impressive piece of technology as a mental metaphor. As exemplars, the ancient Greeks used models of torque-powered siege devices, de La Mettrie's (1960) L'Homme Machine used images of clockwork brains, Freud's libidinous mind was powered by hydraulic instincts, and so on (see Daugman, 2001 for a more extended discussion).

But, as an important historical fact the order of technology-then-metaphor is the other way round in respect of the computational model. Thinking about thinking—specifically Boole's thinking about thinking–came long before the technology did. The technology grew out of it. Thus, it's less true to say that computers are a metaphor for thinking, than that thinking is a metaphor for computation.

One important difference that modern computers have from the “technology as metaphor” pattern is that in none of the other cases have advances been made in the technology as a result of the comparison. Fountains, hydraulics, and clockwork did not become more sophisticated by reflecting on their mind-like properties. On the other hand, artificial intelligence has advanced considerably—to the point where it might be said, without hyperbole, that AI is in many cases the proof that psychology as a science is advancing. When we can formalize an information processing subsystem we can mechanize it. The fact is that we now live in a world where cars drive themselves, airplanes land themselves, and face recognition software finally works.

Deep Mind is living (!) proof that that the Rescorla and Wagner (1972) model of conditional learning works and this is not a unique example (Van Hasselt et al., 2015). The human mind isn't a computer (Searle is right about this) but it does have thousands of computable functions and we are making progress in understanding them. Will there be anything left over when we have solved all these so-called easy problems of Chalmers (1996)? It is too early to say. However, one thing that won't be left over is the ecology. Barrett et al. (2015) have seen to that, by drawing attention to the fact that said functions will be incomplete unless put in ecological (e.g., locally adaptive) contexts. And that's progress, but it is still functionalist progress. Indeed—it's a justly celebrated advance on the Gibsonian programme of embodied functional analysis of cognition. But—it is not less functionalist for all that. It turns out that the details of being an adapted organism (functioning in its ecology) cannot be fully abstracted into discrete disembodied modules fully specificable in terms of brains alone. This might lead some to prematurely think that functionalism has met its nadir, but this would be a mistake. Before I get to why this is I need to say a few things about the Boolean programme that underlies the functionalist revolution in cognitive science.

For an exhaustive exegesis of Boole's work here the authority is (Corcoran, 2003), but the key ideas are quite accessible. Boole's basic insight receives its fullest expression in The Laws of Thought (Boole, 1854) and this is an attempt to draw in all human cognition (it was never about just mathematics) together in terms of the deep underlying logical structure in the most abstract form possible, while still being recognizable at a syntactic level—this level being instantiated (in computers) in terms of logic gates. Formalizing cognition was itself the process which allowed physical computers to be eventually possible.

The major later figures in this development are well known. They include (but are not limited to) Claude Shannon, whose 1947 master's thesis ushered in modern information theory, through Alan Turing whose 1950 paper offered a principled way to instantiate a machine that could compute any computable function (Turing, 1950). John von Neumann's complex proof of how any machine is really a representation of a function (and might thereby replicate itself) was also an important landmark, in von Neumann and Burks (1966). Although all of these papers had important practical outcomes and were (non-accidentally) made by people with engineering connections, they were not “how to build” papers. They were concerned with the formal ways to represent cognition at the most basic level appreciable by human beings. Note that this is not the same as saying that this is the only level they exist at. Those formalizations resulted in physical objects—such as the one I am typing this on—but the causal arrow was not from object to concept. Computers (such as the ones used to crack the Enigma codes) existed by the time of Shannon, Turing, and others but the foundational functionalist work had been done a century before by Boole. Thus, it is strictly illegitimate to say that functionalism, as a strategy for decomposing thought, relies on the computer metaphor. The functionalism came first.

So much for history. Are there independent reasons for thinking that the functionalist programme is not to be lightly set aside? Indeed there are, but here I will only mention a few relevant to Barrett et al. (2015) general programme, which I should stress, are not things that they necessarily deny.

It's commonly asserted that the computational metaphor is about the formal manipulation of symbols (Searle, 1990). But this is a half-truth. At one level, a level that makes semantic sense to a human observer, computers manipulate symbols. But mainly what they do is turn logic gates on and off really fast. And no human observer would be able to make any sense of that at the speeds that it occurs in a modern computer. Of course, if you delve deeper still what we have in the computer is bits of information, and witnessing that wouldn't convey anything much that an unaided human observer could make meaningful. Indeed, the (physical) computer is itself the aid. Boole's key insight was to analyse the logic of human cognition at the mid-level and realize that this level could be formalized. And once something can be formalized it can be mechanized. And the proof that he was right is the tasty pudding of modern computing—which undeniably works, or you would not be reading this.

Does a modern desktop computer (or any computer for that matter) replicate human consciousness? Of course it doesn't. But the formalization of human cognition is a different matter—the computer comes along almost as a by-product of the attempt to do that (albeit a by-product that demonstrates that we must be on to something).

It might be objected that humans do not naturally think in terms of logic gates. And this is true, but hardly to the point. We are typically unconscious of the underlying computational structure of things that come naturally to us. Most of us are unconscious of the grammar of our native tongues unless it is formally taught to us, and it is entirely unnecessary to learn the formal grammar of a language to be able to converse in it. Nevertheless, the formal grammar lays bare the structure of that language.

A follow-up objection might be that, while it is admitted that Boole laid bare the formal elements of some aspects of human thought, there are others left untouched. This may well be true and if it is true then the attempt to build upon his insights with formal instantiations of computation into physical systems that replicate human thought will be forever doomed. Once again—it is too early to tell.

One further common mistake is to note that humans aren't conscious of these sorts of processes. Cognition is not consciousness. Moravec (2000) drew insightful attention to precisely this fact. He noted that the tasks that required very smart humans to perform (e.g., diagnose disease, fly airplanes, play chess) were comparatively trivial to automate (incidentally—this doesn't imply that the automated version completely captures the path of human cognition to achieving them). At the same time, it proved very hard to automate things that to humans were trivial, such as climbing stairs and recognizing faces. The solution to this paradox is that evolutionarily ancient processes do not need to draw on novel conscious elements. But—and this is the crucial point—they are nonetheless cognitive functions for all that.

Computational modeling is rooted in the realization that all observations reveal detectable differences. These are information. If a set of these can be meaningfully grouped into a system then a change is a state change, and any regularities in such changes describe a computational—that is a functional-system. Thus, computation would exist even if computers didn't—this is where critiques like those of Searle's (1990) miss the point. The fact that an existing physical computer is, as he puts it, “just a hunk of junk” is neither here nor there. Once the system can move between states and store them it's a Turing machine, Post machine, or Lambda calculus (Church, 1936)—which for these purposes don't have any significant differences between them. All such functional states are computational states—defined by the moving from one state to another. Knowledge—and it doesn't matter here if we are talking about humans, other animals, or even plants, is therefore the acquiring of usable local regularities. An ecology, in other words. Evolution has produced systems that predict things about their environments (brains) that sometime hang out together in social groups. But all of these things are computational states—and adding ecology to the complete picture does not change this fact. Indeed, it deepens it by showing how affordances must be part of the complete functional picture. Indeed, as Barrett et al. (2014, 2015) are showing, the minimalist bet of some branches of cognitive science—e.g., that we could completely capture the functionalist understanding of the organism without seeing the details of the system it lives in, may well turn out to be false. It turns out that we do need to understand how an organism responds to affordances, that the functional details of perceptual organization matter, and so forth.

But, since we are all functionalists, we really have very good reason to all get along. If it really is functionalism all the way down—then there is no radical split to be had between functional models and the ones Barrett et al. (2015) espouse. What she and her colleagues have done is draw attention to the need for (computational) systems to be closely connected to their ecologies. Specifically, that perception and cognition indeed need to be closely related (Barrett, 2011, p.22). It might be noted that, in this, she echoes the call of Brooks (1990) whose use of the concept of subsumption layers reminds us that one way to escape the representational issue in artificial systems is to make the system use the real world as its model and in this they offer a much needed route to allow affordances to enter into the modeling. Functionalism isn't just the only game in town. It's the only game in any ecology.

## Author contributions

The author confirms being the sole contributor of this work and approved it for publication.

### Conflict of interest statement

The author declares that the research was conducted in the absence of any commercial or financial relationships that could be construed as a potential conflict of interest.
